# Activity recognition with wearable sensors on loose clothing

**DOI:** 10.1371/journal.pone.0184642

**Published:** 2017-10-04

**Authors:** Brendan Michael, Matthew Howard

**Affiliations:** Department of Informatics, King’s College London, London, United Kingdom; University of Illinois at Urbana-Champaign, UNITED STATES

## Abstract

Observing human motion in natural everyday environments (such as the home), has evoked a growing interest in the development of on-body wearable sensing technology. However, wearable sensors suffer from motion artefacts introduced by the non-rigid attachment of sensors to the body, and the prevailing view is that it is necessary to eliminate these artefacts. This paper presents findings that suggest that these artefacts can, in fact, be used to distinguish between similar motions, by exploiting additional information provided by the fabric motion. An experimental study is presented whereby factors of both the motion and the properties of the fabric are analysed in the context of motion *similarity*. It is seen that while standard rigidly attached sensors have difficultly in distinguishing between similar motions, sensors mounted *onto* fabric exhibit significant differences (*p* < 0.01). An evaluation of the physical properties of the fabric shows that the stiffness of the material plays a role in this, with a trade-off between additional information and extraneous motion. This effect is evaluated in an online motion classification task, and the use of fabric-mounted sensors demonstrates an increase in prediction accuracy over rigidly attached sensors.

## Introduction

Advances in the manufacture of microelectromechanical systems (MEMS) has enabled the development of very small form factor inertial sensors, such as accelerometers or gyroscopes [1]. These sensors are sufficiently compact and lightweight (commercial IMUs are millimetres in diameter [2]), to allow for the creation of small measurement systems for monitoring bodily movement, while minimising both physical and visual invasiveness. In comparison to laboratory motion tracking equipment (*e.g.,* cameras or high-accuracy IMUs [3]), these small sensors are very inexpensive [4], ranging between $10 to $200. These facts combined have sparked considerable interest in their use in ‘smart clothing’ (*e-textiles*), whereby MEMS sensors are mounted onto a fabric substrate, such as clothing, for unobtrusive measurement of behaviour [5]. By placing sensors within items of clothing, data can be collected in natural non-clinical environments, such as the home. This provides a sensing platform suitable to long-term data collection studies (*e.g.,* studies lasting periods of weeks or even months). Applications can include the detection and monitoring of movement disorders, for example essential tremor (exhibited by approximately 5% of those over 65 years old [6]), or the progression of neurological disorders such as Parkinson’s Disease (PD) [7]. With the increasing life expectancy rate in developed countries, wearable sensing systems have the promise to provide quantitative movement data for use in the rehabilitation of patients [8], or the detection of disease specific conditions [9]. As these sensors are inexpensive and non-invasive, they can also be used to collect data prior to a diagnosis. For example, in the clinical setting, people ‘at-risk’ of a particular disorder can, by wearing these systems, provide a continuous stream of healthcare progression and recovery data to a clinician, which can be used to both understand disorder progress and provide an early warning.

All wearable sensor systems designed for non-invasive use, whether worn in clothing, strapped to the wearer, or otherwise adhered, suffer from *non-rigid attachment to the body*. Fabric-mounted motion sensors are especially prone to reduced accuracy due to movement of the clothing by external forces (*e.g.,* vibrations, air resistance), compromising their effectiveness for activity recognition and monitoring [10]. The predominant way in which this is dealt with, is to attempt to eliminate motion artefacts by: (*i*) minimising sensor motion with respect to the body (*e.g.,* through use of tight clothing [11], or placing sensors in locations affected minimally by body motion [12]), and (*ii*) applying, for example, de-noising techniques from signal processing [13] or statistical machine learning [14].

**Fig 1:**
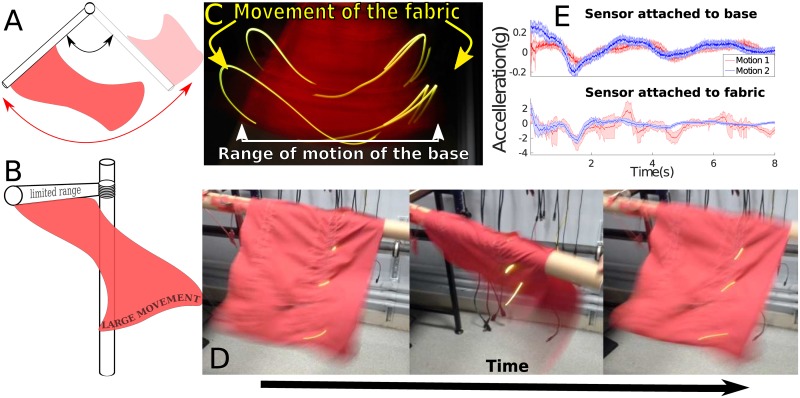
Effects of motion on fabric. The soft, deformable structure of the fabric allows for an increased range of motion **A**, **B**, as seen in LED trails from the top **C**, and side **D** of the fabric. Difference in motion signals in a comparison task **E** where motion sensors are located on both a rigid base, and loosely attached to the base via fabric (note the different vertical scales).

However, this precludes the possibility that *the motion of the fabric itself may also contain valuable information about the wearer’s body motion*. Fabric exhibits features which may, in fact, help in classifying wearer motions, including an increased range of motion and a deformable structure that allows for multi-directional movement, see Fig 1. In the performing arts, this is implicitly exploited for choreographed dance routines: Loose and free-flowing garments are used to exaggerate, emphasise, and express motions to a much larger degree than is possible solely with the human body [15]. Low stiffness materials such as nylon are often used to create a “floating” effect around a dancer, while stiffer fabrics such as jersey grip the wearer [16]. Varying the physical design parameters of textiles to emphasise or suppress particular motion features has also been employed in systems such as vibration isolation (*e.g.,* by varying the knitting method to lower the resonant frequency of material [17]) and for structural deformation (*e.g.,* by introducing auxetic behaviour, whereby a material stretches perpendicular to an applied force [18]).

## Background

To understand how best to exploit such effects in the context of wearable sensing, it is important to understand how the fabric moves when subject to user motion, and how this affects our ability to interpret sensed signals for motion recognition. In the performing arts, choreographers and clothing designers use fabric to best exhibit factors of motion they wish to emphasise. For example, natural materials that easily deform can be used to “catch” the air during motion [19] which allows for a large range of fabric motion from relatively little body motion. This use of the motion of the fabric does not just express the wearer’s body motion on a larger scale (amplification), but selectively emphasises parts of the movement, creating complex motions that the human body could not perform on its own.

If these effects could also be exploited in motion recognition tasks, it may be possible to gain additional information for more robust classifications of movement tasks. To design a *sensing system* that also uses this effect, it is important to understand the role of the fabric parameters (*e.g.,* material properties such as weight) on motion recognition. One way to examine this relationship is to use detailed models of the fabric structure, including the behaviour of individual yarns [20], to simulate interaction dynamics between wearer and fabric [21]. However, such simulations are computationally expensive (requiring several seconds of computing time for individual animation frames [22]), and are of questionable accuracy due to the difficulty in determining fabric parameters (*e.g.,* weave, thread tension, worn position) and external factors (*e.g.,* wind, humidity).

Alternatively, one can directly analyse data from a physical fabric system and use statistical learning methods to examine the effect of varying specific elements of the fabric structure. In a wearable sensing system, this can be used to both create models of the user’s motion from sensed movements, and quantify recognition improvement when varying fabric designs. By statistically quantifying this accuracy, it can be shown explicitly which fabric parameters have the greatest influence in emphasising motion.

**Fig 2:**
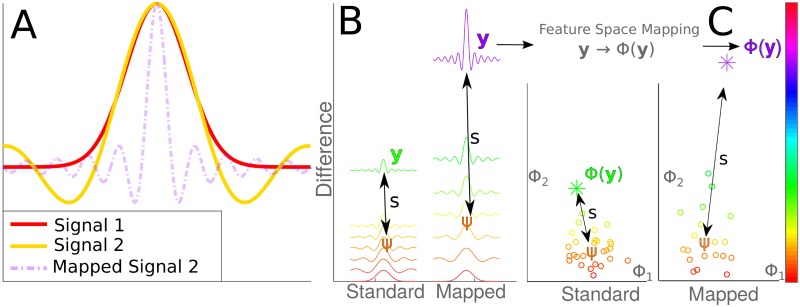
**A** Illustration of how two visually similar functions can be difficult to classify (signal 1 is the Gaussian function with *μ* = 0, *σ* = 2, and signal 2 is sinc(*x*)). These signals can be more easily distinguished when one or the other signal is non-linearly transformed (Mapped Signal 2, is sinc(4*x*))). **B** Visual illustration of the similarity *s* between a cluster mean *ψ* of one signal, and a new signal **y**, for both the normal and mapped systems. **C** Similarity when representing signals as points in a cluster classification system.

By far the dominant approaches to activity recognition in wearable sensing systems are distance-based statistical methods [23] such as K-Nearest-Neighbours or Support Vector Machines (SVM) [24]. In this setting, the sensed movement (*e.g.,* the sensed unidirectional acceleration of an arm during a reaching task) is recorded and represented as a fixed length vector $y\in R^{P}$, where $y:={\left(y_{1},y_{2},\ldots ,y_{P}\right)}^{T}$, and *y*_*t*_ is the sensed reading at time *t*. In classification systems, these sample movements are then represented as points in some feature space $\phi \in R^{J}$, and the contrast between these points is used to determine the class label, see Fig 2.

The key to success in such approaches is the selection of the feature space *ϕ*, which plays an important role in building good predictors [25]. To find features in the data that may result in highly accurate classifiers, processes such as *feature extraction* or *dimensionality reduction* can be used. For example, principle component analysis [26] is often used to decompose a data into a set of linearly uncorrelated variables, allowing for the removal of variables that only contribute minimally to describing the data. These features can also be selected a priori, for example in myopathic studies, decomposing raw electromyographic signals into frequency domain information often results in highly significant differences between healthy and myopathic patients, increasing the accuracy when diagnosing neuromuscular disorders [27].

In the context of fabric-based sensing systems, the fabric itself can be thought of as a feature space transformation from the wearer’s body motion. This transformation is defined as the one-to-one mapping $\phi \left(y\right):R^{J}\mapsto R^{J}$ between the sensed motion from the body and that of the fabric (note that in this approach, J=P). In other words, fixed-length motions **y** of a given type are collected and transformed into the feature space *ϕ* = *ϕ*(**y**). Note that, by using the fabric as the feature space transformation, the transformation is obtained simply by placing sensors *on the fabric itself*, requiring no additional processing.

To compute a classification model, M samples of motions *ϕ* in the feature space are used to form a cluster $\Psi \in R^{P\times M}$, from which a single model representing the full data set is derived $\psi \in R^{P}$ (*e.g.,* the mean of the cluster). The *similarity s* between *ψ* and a new feature-space-mapped motion sample *ϕ*′ scores the extent to which the latter belongs to this cluster of motions. This is computed as the distance *s* = *d*(*ψ*, *ϕ*′) according to some chosen metric (*e.g.,* the Euclidean or Mahalanobis distance [28]). A small value of *s* indicates that the new motion sample *ϕ*′ is a member of the cluster **Ψ**, while a large value (a large *dissimilarity*) indicates that *ϕ*′ is a movement of a different type.

The present study empirically investigates whether varying the structure of a fabric-based sensing system increases motion prediction accuracy due to selective emphasis of parts of the motion. A statistical approach is taken whereby the physical fabric motions are analysed through the statistical classification techniques described above.

**Fig 3:**
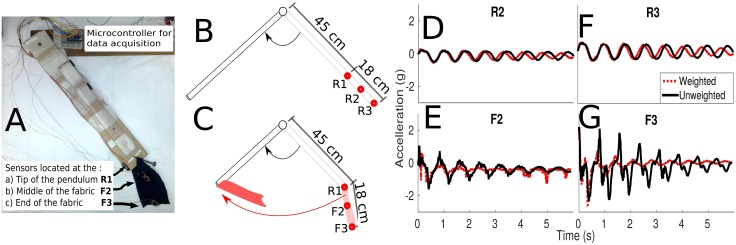
**A** Side-view of pendulum with attached fabric at tip. Sensors are placed at the tip of the pendulum, and on the body of the fabric. **B, C** Schematic of experimental setup. **D, F** Motion signals from both weighted and unweighted pendulum when sensor is attached rigidly to the pendulum at increasing distances from the axle. **E, G** Motion signals when sensor attached to the end of the fabric.

For this, the data acquisition device shown in Fig 3(A) is used. In the experiments, motion signals are recorded, varying factors of the experimental setup, including types of fabrics used and similarities between motions. Given these motion signals, the *similarity* between types of motion is analysed, to determine which factors of the fabric influence motion prediction accuracy.

## Materials and methods

### Data collection

The data acquisition device (Fig 3(A)) used in this study consists of a weighted pendulum (of length 57 *cm*), swinging freely in gravity on a single axis.

A fabric substrate (heavy weight jersey, two-way stretch, 95% cotton, 5% elastane, 18 *cm* length when taut), is attached to the tip of the pendulum. The device is instrumented by three inertial sensors (LilyPad ADXL335 tri-axial accelerometer) that simultaneously record the acceleration of different points on its length. These sensors are attached at (*i*) the tip of the rigid pendulum (denoted R1, 57 *cm* from the pivot), (*ii*) in the centre of the fabric (F2, 66 *cm*) and (*iii*) at the tip of the fabric (F3, 75 *cm*).

The accelerometers are sampled using an Arduino Nano (Atmega-328P microcontroller, 16-bit ADC), and readings are transferred to an attached PC base-station for analysis. The accelerometers are connected to the microcontroller by loose, thin, light-weight insulated copper wiring to ensure minimal interference with the motion of both the pendulum and fabric. All sensors are calibrated to one-another to remove inter-sensor variability, and collect data at 600Hz.

In each recorded motion, the pendulum is released from a static position (the horizontal), and data is recorded from all three sensors for 10 seconds. External environmental factors, such as vibrations and air currents, were minimised by performing the experiments in a closed laboratory. During motion, acceleration signals are collected from the axis parallel to the ground (when the pendulum is at rest) from all three sensors. Two sets of data are collected, in the first the pendulum is weighted with 3*N* at the tip, and in the second the weight is removed. This process of data collection is repeated to produce 10 independent motions per data set. All motion signals are converted to standard gravity, then time-synchronised. For each data set, a cluster of the 10 motions recorded over 6000 time-steps is then defined as the cluster matrix $\Psi \in R^{6000\times 10}$, and the mean $\psi \in R^{6000}$ is taken to form the model of the motion.

### Varying fabric material

To evaluate how the structure and physical properties of the fabric can influence the similarity score, the same experiment is repeated with several other commonly-used clothing materials, denim (98% cotton, 2% elastane), jersey, and roma (four-way stretch 74% polyester, 27% rayon, 3% spandex). The process is also repeated with the fabric replaced by a rigid element, with sensors R2 and R3 placed at identical distances to the pivot as F2 and F3.

### Varying pendulum weight

To examine the effect of the motion itself, the experiment is repeated using the jersey material with pendulum weights varied between 0.5*N* to 3*N*. This variation alters the speed of the pendulum, thereby generating different motion signals. In the context of wearable sensors monitoring human motion, this simulates the common task of a wearer performing tasks with different speeds (e.g. lifting weights for rehabilitation exercises [29]).

### Statistical analysis

To examine the similarity between different motions, these experiments compute the Euclidean distance between signals *ϕ*, normalised over the range of distances. One-way analysis of variance is performed using the Matlab R2016b statistics toolbox. Depending on the experiment, input data is either motion signals $\phi \in R^{6000}$ or similarity scores *s*, and data is grouped according to their weight.

### Motion classification

To perform classification and prediction, classification methods are trained using the data collected from the fabric tipped pendulum, to predict whether the pendulum is swinging with or without a weight attached. In these experiments, support vector machines (SVM), (two-class, using the Matlab R2016b statistics and machine learning toolbox) and discriminative regression machines (DRM) [30] are used. For both competing methods, linear [24] and Gaussian kernels [31] are used, with the hyper-parameters *box-constraint* and *kernel scale* obtained via a five-fold cross-validation. Note that this implementation can be extended to the multi-class setting by using common ensemble techniques such as “one-vs-one” or “one-vs-all” [32].

For each of the three sensors, 10 samples of pendulum motion with a 3N weight and 10 samples without, are randomly segmented into two independent sets, a model training set consisting of 19 motion samples and an independent testing set consisting of one sample.

As is common in wearable prediction systems that work from continuous streams of data [33] (i.e. where it is not known when one motion finished, and another begins), an online learning and prediction method is used. In this, time-discrete segments of the motion signal are passed to the classifier as they are recorded. In this study, motion signals *ϕ* are segmented into *windows* of size *n* (where n<P). A small value of *n* signifies that only a small segment of the temporal motion signal is used to compute classification models and make predictions. As *n* increases (i.e. *n* → ∞), more of the motion signal is used. Windows overlap by every $\frac{n}{2}$ time-steps, with predictions made every $\frac{n}{2}$ time-steps. These windows can be defined as subsets of the full motion signal,

$$\begin{array}{c} w:=[{\left(\phi_{1},\ldots ,\phi_{n}\right)}^{T},{\left(\phi_{\frac{n}{2}},\ldots ,\phi_{\frac{3n}{2}}\right)}^{T},{\left(\phi_{n},\ldots ,\phi_{2n}\right)}^{T},\ldots ] \end{array}$$

To perform model training and prediction, initially the first *n* time-steps of the training data are used to train a classification model, and predictions are then made on the first *n* time-steps of the testing data. The window is then time-shifted forwards by $\frac{n}{2}$ time-steps, and process is repeated. This repeats until the end of the signal is reached. To ensure robustness of the modelling method, this online learning and prediction method is repeated 20 times, varying the motion sample used for the testing set.

With this experimental setup, the effect of the window size used to segment the motion data is examined, by evaluating the classifier accuracy for each sensor when varying the size of the window between 15 milliseconds, and 1.5 seconds. From this evaluation, a fixed window size is selected to examine the normal operation of the classifier.

## Results

### Effect on sensed motion signal

To examine how sensed readings from fabric-embedded sensors can be exploited in wearable sensing systems, this section examines if significant differences between similar pendulum motions can be observed.

Fig 3(D) shows two motion signals from a trial generated by the apparatus, collected from sensor R2, with the pendulum weighted with 3*N*, versus the unweighted pendulum. The addition of this small weight causes a change in motion of the pendulum, shown by the introduction of a phase offset over time. In Fig 3(F), the motion signal from sensor R3 (located further along the pendulum) is plotted.

It is seen that, as expected, increasing the distance from the axle increases the amplitude of the acceleration signal. This is due to the geometry of the set up; sensor signals have larger amplitude with increased distance from the pivot (linear acceleration of the tip increases with pendulum length). To examine if the sets of weighted and unweighted signals are significantly different from each other, motion signals $\phi \in R^{6000}$ from each sensor is allocated to groups according to their weight. It is seen that there is no significant difference between weighted or unweighted motions observed either from sensors R1, R2 or R3 (*p* > 0.75). This shows that in this setup, attempting to predict if a motion is weighted or unweighted by using rigidly attached sensors, is a challenging task.

It is seen in Fig 3(E) and 3(G) that when the lower two sensors are mounted onto fabric, there is a much larger difference between the two signals compared to that seen with a rigid extension. The difference between motions is most noticeable in readings from the sensor mounted furthest from the fabric attachment point Fig 3(G), where different oscillatory patterns emerge between the motions due to changes in direction of the pendulum causing secondary swinging motions of the fabric. There is a significant difference between the two motions, for both sensors F2 and F3 (*p* < 0.01). From this, it can be seen that there are significant differences between motions, but only observed when sensing the motion of the attached fabric.

### Effect on classification algorithms

To examine how these observations affect distance based classification algorithms, this section examines if contrasting motion signals observed from fabric-mounted sensors show greater *dissimilarity* than rigidly attached sensors. Greater dissimilarity between motions is increased by the enhancement of contrasting features, and is the underpinning factor in many commonly used classification algorithms. To examine this, the following experiments examine the similarity between the two sets of motions.

**Fig 4:**
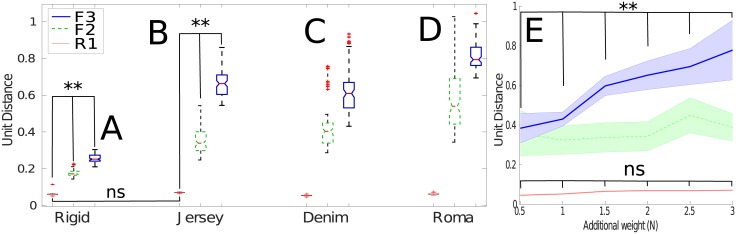
**A-D** Box plots of the difference between unweighted and weighted pendulum when using sensors located at the pendulum tip (red thin line), middle (green dashed), or end (blue thick line). **E** Difference for jersey when varying weights from 0.5*N* to 3*N*.

Initially when observing motion signals from the rigid pendulum (Fig 4(A)), it is seen that the average similarity between the weighted and unweighted pendulum signals decreases as distance from the axle increases (p<0.01). However, this difference between motions is small, for all three sensors located rigidly on the pendulum. In comparison to this, there is a much larger difference between motion signals when observations are recorded using fabric sensors mounted onto jersey (Fig 4(B)). The greater distance between the two motions when using jersey indicates that the signals observed from fabric-mounted sensors are not only less similar (and thereby easier to distinguish in a classification system), but are more robust to in-class variance in the sensed readings, increasing the *confidence of the predictions*. The signals from sensors placed at the middle and end of the fabric also show more variance due to external environmental factors (*e.g.,* air movement, small variations in starting angle) causing greater spread in the data cluster.

One possible explanation for the difference between motions, is that the fabric significantly *alters* the motions of the pendulum (e.g. by increasing the air resistance and slowing down the pendulum). To evaluate this, similarity measurement are taken from the signals recorded from sensor R1, which is attached rigidly to the pendulum during all experiments (including fabric-mounted ones). In this, it is shown there is no significant difference (p>0.9) in the similarity measures when using either a rigid pendulum or with a jersey tip. This indicates that the increase in distance is not caused by the additional fabric biasing the signal by altering the underlying pendulum motion.

### Effect of varying pendulum weighting

The similarity between motions when varying the pendulum weight (Fig 4(E)),shows a similar trend: the average similarity score between motions for the sensor located on the pendulum (R1) shows no significant difference (*p* > 0.50) across conditions (pendulum weights). For the sensor located in the middle of the fabric (F2), the average similarity varies slightly between conditions. As the centre of the fabric undergoes less deformation than the end of the fabric (F3). At the end of the fabric there are significant differences across conditions (p<0.01). These experiments show that while there are no significant differences between the motion when observing from the rigidly attached sensors R1, differences in the similarity between motions can be seen when using fabric.

### Fabric structure and the relationship to similarity

In experiments varying the fabric material used, sensors placed on both the denim Fig 4(C) and roma Fig 4(D) result in a larger dissimilarity between motion classes than the rigid pendulum. It is seen that denim shows greater similarity than jersey or roma, due to the rigidity of denim damping the signal, and reducing the oscillatory effects of the fabric in motion. In contrast, the dissimilarity of the sensed motions from the sensor placed on the roma fabric is greatest (with the largest variance), due to the light, non-stiff structure of the fabric.

### Activity recognition

In a classification setting, the above suggests the surprising result that it should be *easier* to distinguish between different motions with a sensor mounted onto clothing, than one rigidly connected to the body. To evaluate this, a classifiers are used to predict whether the pendulum is swinging with or without a weight attached, using the data collected from the jersey tipped pendulum. In a motion recognition context, the sensors can correspond to sensors mounted on a loose area of a garment, such as a sleeve. In this context, sensors F2 and F3 correspond to mounting sensors at varying positions of the sleeve, while R1 (which is attached rigidly to the body) simulates a body worn sensor. Classification is performed using the traditional linear kernel, due to its simplicity in computing classification models (making it suitable for an embedded low-cost system), as well as its straightforward interpretation as a distance-based classifier. Models are also computed using the Gaussian kernel due to its common usage in activity recognition [34] and the discriminative regression machine (DRM), due to its suitability in dealing with similarities within classes of high-dimensional data with small sample sizes [30].

### Effect of window size

**Fig 5:**
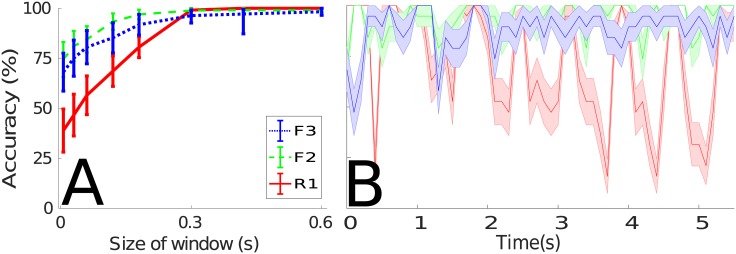
Mean accuracy of Gaussian SVM over 20 trials (± standard error). **A** when varying window size between 15*ms* and 1.5 seconds, and **B** mean accuracy of online motion prediction with window size fixed at 250*ms*, using data from the sensors attached to the pendulum and fabric. Similar results for both experiments are also seen for linear SVM, and both linear and Gaussian DRM.

Using the online classification system, the results for varying the size of the window are shown in Fig 5(A). Initially, it is seen that at a window size of 15*ms*, the prediction accuracy from the rigidly attached sensor (R1) is approximately 40%, while the fabric-mounted sensors report accuracies of 70% and 75%. As the size of the window increases, the prediction accuracy also increases, as expected due to the additional information of the motion signal available to the classifier. The fabric-mounted sensors continuously predict at a greater accuracy than the body-mounted sensor. As the size of the window surpasses 300*ms*, the body-mounted sensor predicts motions with 100% accuracy, while the fabric-mounted sensors F2 and F3 predict at 95% and 90% respectively. This result indicates that at small window sizes, where predictions can be made more rapidly with fewer computations, fabric mounted sensors outperform their body-worn counterparts. At larger window sizes, where there are greater time-periods between predictions, the fabric-mounted sensors fall slightly short of the accuracies reported by the body-worn sensors.

### Online prediction

Closer evaluation of the classification results at a fixed window size of 250*ms* is shown in Fig 5(B). In this, it is seen that on average the prediction accuracy using SVM from the rigidly attached sensor is 83.9 ± 17.9% (linear kernel) 84.1 ± 20.6% (Gaussian kernel), while the average accuracy from the sensor located in the middle of the fabric is 93.3 ± 8.6% (linear kernel) 95.9 ± 6.1% (Gaussian kernel). Predictions made using the DRM classification method, give accuracies from the rigidly attached sensor of 72.4 ± 1.7% (linear kernel) 80.3 ± 18.1% (Gaussian kernel), and for the sensor located in the middle of the fabric 90.6 ± 9.1% (linear kernel) 94.5 ± 6.7% (Gaussian kernel).

These results show that not only is the average prediction accuracy higher for the fabric-mounted sensors, but the lower variance in the predictions indicates that the classifiers used are more robust. This follows on from the similarity analysis (see above), which shows that the greater distance between types of motion signals from fabric-mounted sensors results in a greater confidence when making predictions. It should also be noted that the body-mounted sensors (Fig 5(B)) demonstrate oscillating dips in prediction accuracy. This is due to pendulum moving in and out of phase between the weighted and unweighted motion signals (*e.g.,* at the apex of a swing, where the recorded acceleration is zero), resulting in high similarity between the signals. In contrast the fabric does not exhibit this effect, as the deformable structure of the fabric allows for complex movement in other axes, resulting in motion trajectories that are significantly dissimilar, at similar positions of the pendulum. The similar results seen in all three classification methods demonstrate that the exploitation of the fabric dynamics plays a greater role in achieving high accuracy, than the complexity of the classifiers used.

## Discussion

In this paper, an empirical investigation into the the use of fabric-mounted sensors has been performed, to examine if the non-rigid, deformable nature of fabric can be exploited to provide additional information about a wearer’s movement, enhancing activity recognition systems. The findings outlined here show that mounting MEMS inertial sensors onto loose fabric can lead a greater contrast between different types of measured motion. Even when signals from a rigidly attached sensor are not significantly different, the fabric’s ability to deform in multiple directions allows for an increased range of motion, making it easier to distinguish between different motions. In motion classification tasks using streaming data, the use of fabric mounted sensors can result in a greater prediction accuracy, with smaller windows of data, allowing predictions to be made more quickly and at lower computational cost compared to the rigidly attached equivalent. This high accuracy coupled with a relatively simple linear SVM classification algorithm, demonstrates that this approach of exploiting the dynamics of the fabric can enhance this classification accuracy with no need for greater computational power, even in comparison to using learning methods which explicitly account for high dimensional data and small data sets.

The effects seen in this paper depend on factors such as the speed and amplitude of the motion and the material properties of the fabric such as its length and stiffness. However, it is robustly reproduced in a number of fabrics commonly used in ordinary clothing. Fabrics with a low stiffness flow more easily, subjecting sensors to greater accelerations. However, this low stiffness also means that the fabric motion is more sensitive to environmental factors (*e.g.,* air flow), and results in larger within-class variance. There is a trade-off between using motion artefacts to emphasise selective features, and limiting the effect of motion artefacts on the predictions. This is especially important when applying this method to real-world motion tasks, where poor control of this trade-off may result in highly unpredictable motion artefacts, masking the intended signal. Nevertheless, the experimental results on integrating sensors into deformable materials presents the first evidence that *noise and motion artefacts can be beneficial to motion recognition tasks*. With the advent of modern, model-free statistical approaches to activity recognition, requiring that motion artefacts always be eliminated is not only unnecessary, but *fails to exploit information implicit in the textile motion*.

In the context of real-world activity recognition, this method in its current form would find utility in classifying controlled motion tasks (e.g. rehabilitative weight-lifting exercises for muscle strengthening [29]). This approach shows a promising baseline for future work involving unconstrained human motion. The exploitation of soft sensor deformation has wider implications outside the field of sensorised clothing. The observations reported here may also find utility in other soft sensing based applications, *e.g.,* healthcare monitoring devices such as sensorised mattresses for measuring cardiac and respiration during sleep [35], or capacitive textile sensors in car seating to capture whole body motion to detect impaired driving [36]. Not only would the ability to make sensitive predictions enhance current applications, but the revised view of utilising motion artefacts enables the development of systems previously thought to be too noise-corrupted.
